# Interactions Between Corn Starch and Ethyl Maltol Under Heat-Moisture Treatment and Its Application in Fried Chicken Nuggets

**DOI:** 10.3390/foods13223629

**Published:** 2024-11-14

**Authors:** Meijuan Xu, Tianwen Liu, Xueqin Gao, Yuran Shi, Xiaodong Zhao, Jian Zou

**Affiliations:** 1 College of Food and Biological Engineering, Henan University of Animal Husbandry and Economy, Zhengzhou 450046, China; xumeijuan113@163.com (M.X.); kfgxq03@163.com (X.G.); shiyuran0907@163.com (Y.S.); 15093306674@163.com (X.Z.); 2 College of Food Science and Technology, Huazhong Agricultural University, Wuhan 430070, China; 3 School of Life and Health Sciences, Hubei University of Technology, Wuhan 430068, China; ltw15515757402@163.com

**Keywords:** heat-moisture temperature, non-inclusion complex, structure, coating powders

## Abstract

This study delved into the interaction between corn starch and ethyl maltol during innovative repeated continuous heat-moisture treatment (RCHMT) and its impact on the quality of fried chicken nuggets. The results reveal that the complexation ratio of ethyl maltol is about 31.6%, and the complex creates dense microporous structures. Native starch and complex samples exhibited an A-type crystal structure, while the physical mixture sample showed superposition peaks of starch and ethyl maltol. Additionally, the peak of C-O-H def., CH_2_ of the complex sample was blue-shifted to the larger wave number, and the hydrogen bond structure was enhanced. Moreover, the complex exhibited a higher resistant starch content and lower hydrolysis rate and amylose content than the physical mixture sample. The starch–ethyl maltol complex has been demonstrated to be a non-inclusion compound. It has been shown to reduce oil absorption and enhance the crispness of fried chicken nuggets, matching that of commercial products. This finding provides a direction for the development of innovative coating powders.

## 1. Introduction

Deep-fried products have gained popularity due to their desirable golden color, satisfying crispy texture, and unique flavor [1]. In particular, fried coated products, such as chicken nuggets and small crispy meats, have demonstrated remarkable growth in recent years. However, excessive oil absorption during frying and cooling creates a greasy texture and an unfavorable consumer experience [2]. High-oil-absorption products contribute to obesity, cardiovascular disease, and other health issues [2]. Moreover, industrial frozen meat in the thawing process causes flavor loss, resulting in fried-coated food products lacking a meaty flavor [3]. Simultaneously, the reduced crispiness and quick loss of texture are the non-negligible challenges of deep-fried coated food products, which are attributed to the starch type in the coating, the frying process, and freezing procedures [4,5]. Researchers have explored various innovative strategies to address these challenges, such as enhancing crispiness, reducing oil absorption by cross-linked and pregelatinized modified starches [6,7], and improving meat flavor stability by introducing ethyl maltol. Nevertheless, the structure of the cross-linked and pregelatinized modified starches was severely destroyed [7], which limits the further modification of starch. The high-temperature instability and extreme volatility limit ethyl maltol’s application [8]. Under the premise of maintaining the starch’s structure, it is important to develop a physical modification technology that combines starch with ethyl maltol, which not only improves the stability of flavor substances, but also produces starch with high crispness and low oil-absorption properties.

During food processing, starch forms complexes with ligands such as fatty acids, polyphenols, and flavor compounds [9]. Starch granules swell as they absorb water, causing starch chains to open and twist into hydrophobic helical voids through helical folding under hydrogen bonding, with hydrophobic methylene and glycosidic bonds in the interior and hydrophilic hydroxyl glucose residues on the outside [10,11]. Small ligands entering the helical structure of amylose form V-type crystal structures or left-handed single helical complexes [10], while larger ligands form complexes through hydrogen bonding on the outside of amylose chains [11]. The interaction between starch and ligands relies on non-covalent bonds, such as hydrogen bonding, hydrophobic interaction, CH-π bonding, etc. [10,12]. The hydrogen bonding and hydrophobic interactions within the system are crucial in facilitating the complexation process between starch and ligands, leading to significant modifications in the rheological and digestive properties of the starch [13].

Wall materials containing flavor substances include cyclodextrins, starches, hydrophilic colloids, and proteins. Among them, starch is the most inexpensive and effective method for encapsulation. Methods for preparing starch small-molecule flavor substance complexes mainly include dimethyl sulfoxide solvent, alkali, zinc chloride, hydrothermal, and ultrasound-assisted preparation methods. The alkali method and zinc chloride solvent method promote the transformation of the starch’s helical structure into a random coiled shape, thereby enhancing the binding of the helical structure with thymol to form a V-type crystal structure [14]. Still, the chemical methods introduce chemical reagents, contradicting the development trend of “clean label”. Hydrothermal methods and ultrasound-assisted hydrothermal methods are significantly affected by hydrothermal conditions, and strict control of water content and processing temperature is required to complex with the ligand [15,16].

Heat-moisture treatment is a form of hydrothermal physical modification technology. The repeated heat-moisture technique involves processing starch through a cyclical process of heating followed by cooling, while the continuous heat-moisture technique entails subjecting starch to uninterrupted heating for a specified duration. Our previous research indicated that repeated heat-moisture treatment can effectively open the helical structure of starch by utilizing the temperature differentials generated during heating and cooling, and continuous heat-moisture treatment can facilitate the rearrangement of amylose and amylopectin and enhance the crystalline structure of starch [17,18]. Building on this foundation, the present study hypothesizes that repeated heat-moisture treatment may facilitate the opening of starch’s helical structure, thereby exposing additional hydrogen bonding sites and promoting the interaction between ethyl maltol and starch through non-covalent or covalent bonding. Ultimately, continuous heat-moisture treatment is employed to further rearrange the molecular chain structure of starch, enhancing its structural integrity. Consequently, a starch–ethyl maltol complex sample was prepared by repeated heat-moisture treatment and continuous heat-moisture treatment (RCHMT), allowing for an analysis of their complex interactions under this methodology. A correlation between structural characteristics and digestibility was established. Finally, this complex sample was applied to fried, coated meat products to discuss its impact on chicken nugget quality.

## 2. Materials and Methods

### 2.1. Materials

Normal corn starch (purity ≥ 98%) was purchased from Nanjing Huafeikou Alkali Factory Co., Ltd. (Nanjing, China). Ethyl maltol (purity ≥ 99%) and α-amylase (11 u/mg) were obtained from Yuanye Biotechnology Co., Ltd. (Shanghai, China). Glucosidase (10^5^ u/mL) was purchased from Maclin Biochemical Technology Co., Ltd. (Shanghai, China), and a D-glucose test kit was purchased from Megazyme Co., Ltd. (Wicklow, Ireland). Methanol (GC analysis, ≥99.9%) was from Innochem Technology Co., Ltd. (Beijing, China). Other chemical reagents were analytically pure and sourced from Guoyao Co., Ltd. (Beijing, China). Commercially available chicken nuggets were provided by CP GROUP.

### 2.2. Preparation of Sample and Determination of Structure and Digestibility Properties

#### 2.2.1. Preparation of Corn Starch–Ethyl Maltol Complex and Mixture Sample

The factors affecting the complexation ratio of the corn starch–ethyl maltol complex prepared by RCHMT were excavated through single-factor and response surface tests. Factors, such as the water content (factor 1), amount of ethyl maltol (factor 2), treatment temperature (factor 3), cycles of repeated heat moisture (factor 4), and treatment time of continuous heat moisture (factor 5), were identified. This result is shown in the Supplementary File.

The meticulous process of recombination was as follows: The weighted 100.00 g of native corn starch (named for native starch) was accurately weighed and the moisture content was adjusted to 34% with distilled water (factor 1). A portion of the distilled water was used to dissolve 0.40 g of ethyl maltol (factor 2). The mixture was then evenly combined in a blue-capped silk bottle and left to balance at room temperature for 24 h for subsequent RCHMT. The sample was heated in an oven at 94 °C (factor 3) for 4 h and allowed to cool naturally at room temperature (25 ± 2 °C) for 1 h. This step was repeated twice (factor 4) to obtain the repeated heat-moisture treatment sample. Subsequently, the abovementioned samples were continuously treated in the oven at 94 °C for 12 h (factor 5) to produce the RCHMT sample. The obtained sample was washed with ethanol-water (1:1, *v*:*v*) three times to remove the free ethyl maltol. Finally, the sample was dried at 45 °C and crushed through a 100-mesh sieve to obtain the corn starch–ethyl maltol complex (named for the complex sample). The mixture sample before RCHMT was used as the control (named for the mixture sample).

#### 2.2.2. Complexing Ratio

The content of ethyl maltol was determined by high-performance liquid chromatography (K2025, Hanon Group Inc., Dezhou, China), according to GB 5009.250-2016. Mobile phase A consisted of methanol, and mobile phase B comprised a sodium dihydrogen phosphate solution. The complex (2 g) was placed into centrifuge tubes, and 5 mL of distilled water and 15 mL of methanol solution were added to each tube for ultrasound treatment for 20 min. Moreover, the different ultrasonic times (1 h, 4 h, 8 h, 12 h, and 24 h) were investigated to evaluate the final released ethyl maltol content of the complex with a 500 W ultrasonic power. The methanol solution was used to keep a constant volume of 25 mL. The solution was centrifuged (6000 r/min) for 10 min. A microporous filter membrane filtered the supernatant. The complexation ratio was the ratio of released ethyl maltol content to total ethyl maltol content.

#### 2.2.3. Amylose Content

The amylose content was quantified using this methodology from Ge et al. (2020) [19] with a slight modification. Fifty milligrams of starch were accurately weighed and placed into a 50 mL centrifuge tube. Subsequently, 0.5 mL of 95% ethanol was added to ensure the complete dispersion of the samples. Following this, 4.5 mL of 1 mol/L NaOH was introduced, and the mixture was vortexed thoroughly before being heated in boiling water for 10 min. The solution was transferred to a volumetric flask with a final volume of 50 mL, followed by vigorous shaking to achieve homogeneity. Next, the abovementioned solution (2.5 mL), acetic acid (1 mol/L, 0.5 mL), and potassium iodide (2% KI + 0.2% I_2_, 1 mL) were added to a volumetric bottle to a total volume of 50 mL with distilled water. Finally, the solution was allowed to stand at room temperature for 10 min before measurement at a wavelength of 620 nm (UV-1500, Macylab Instrument, Shanghai, China).

#### 2.2.4. Scanning Electron Microscope (SEM)

Starch samples were mounted on circular aluminium stubs with double sticky carbon tape, coated with a thin gold film. The morphology of the samples was observed using a scanning electron microscope (SEM, S-3400, Hitachi, Tokyo, Japan) [20]. It was operated at an accelerating voltage of 5.0 kV.

#### 2.2.5. X-Ray Diffraction (XRD)

The crystal structure of starch was characterized using an X-ray diffractometer (XRD, D/MAX2200 pc, Rigaku Corporation, Tokyo, Japan) at 40 kV and 40 mA according to the method of Gong et al. (2017) [21]. A precise sample was strategically positioned and continuously scanned on a rectangular aluminum sheet. The tests were conducted employing a copper target, ensuring accuracy and thoroughness within a range of 5° to 50°, at a speed of 6°/min with a step size of 0.02°.

#### 2.2.6. Fourier Infrared (FT-IR)

Short-range order was assessed based on the method with a slight modification [18]. 

A Fourier-transform infrared (FT-IR, WQF 530) from Beijing Beifen-Ruili Analytical Instrument Co., Ltd. (Beijing, China), was employed. The sample and KBr were mixed in a ratio of 1:50, ground to a fine powder in a mortar, and then compressed into tablets for determination. The measurement was carried out within the range of 4000 cm^−1^ to 400 cm^−1^, with 32 scans and a resolution of 4 cm^−1^. In addition, the Lorenz curve function was deconvolved for the 1200–800 cm^−1^ region using OMNIC software version 8.2, with a half-peak width of 32 cm^−1^ and a factor of 1.9.

#### 2.2.7. ^13^C Solid-State Nuclear Magnetic Resonance (^13^C-NMR)

The sample was placed in a rotor and analyzed using a 599.7 MHZ wide-cavity solid superconducting NMR spectrometer (JNM-ECZ600R/S1, Nippon electronic company, Tokyo, Japan) with a 7 mm MAS BB/BB probe at an MAS speed of 12 kHz. Origin software version 8 was employed to fit the peaks in chemical shifts of 93–107 ppm using Gaussian and Lorentz functions. The correlation coefficient (r^2^) of the fitting must be greater than 0.98.

#### 2.2.8. Differential Scanning Calorimetry (DSC) 

The thermal parameters were assessed according to the method of Ji et al. (2023) [22] with a slight modification using a Differential Scanning Calorimeter (DSC, Q2000, TA Instruments, New Castle, DE, USA). For the DSC analysis, 3 mg of the sample was evenly spread in an aluminum dish and mixed with 9 μL of ultra-pure water. After tightly sealing the dish, it was kept at room temperature for 24 h to achieve equilibration. The temperature was then measured with a heating rate of 10 °C/min within the range of 30 °C to 150 °C.

#### 2.2.9. Digestive Properties 

According to the method of Zou et al. (2023) [20], 50 mg of starch and 10 mL of phosphate buffer (0.5 mol/L, pH = 5.2) were thoroughly mixed and then incubated at 37 °C for 10 min. After that, 4 mL of porcine pancreatic alpha-amylase (3000 U/mL) and 1 mL of starch glucosidase (2500 U/mL) were added to the starch paste. The mixture was then incubated at 37 °C for 0, 10, 20, 30, 60, 90, 120, 150, and 180 min. After each time point, 0.5 mL of Na_2_CO_3_ (0.3 M) was added to the hydrolysate to deactivate the enzyme. Following centrifugation at 5000 r/min for 10 min, 0.1 mL of the supernatant was combined with 3 mL of GOPOD reagent for glucose content analysis. The glucose content at 20 and 120 min was utilized to calculate the rapidly digestible starch (RDS), slowly digestible starch (SDS) and resistant starch (RS).

RDS (%) = (G_20_ − G_0_)/W×0.9×100

SDS (%) = (G_120_ − G_20_)/W×0.9×100

RS (%) = 1 − RDS − SDS

#### 2.2.10. First-Order Kinetic Fitting

The digestibility can be fitted to first-order kinetics according to the method of Zou et al. (2023) [20]. The first-order kinetic equation is:

C_t_ = C_∞_ (1 − e^(−kt))

where:

C_t_ (%): the percentage of starch digested in time t (min);
C_∞_ (%): the estimated percentage of starch digested at the end of the reaction;
k (min^−1^): digestion rate coefficient, estimated by transforming the equation using the slope analysis (LOS).

ln(dc/dt) = −kt + ln(C_∞_k)

where ln(dc/dt) represents the logarithm of the slope, and this equation describes the linear relationship between LOS and starch hydrolysis time.

### 2.3. Application of Complex to Refried Chicken Nuggets

#### 2.3.1. Preparation of Coating Powder

Preparation of original coating powder: wheat flour:potato starch:sweet potato starch = 1:5:5 (*w*/*w*/*w*).

Preparation of composite coating powder: The original coating powder was replaced with the corn starch–ethyl maltol complex in varying proportions (0%, 3%, 6%, 9%, 12%, and 15%).

#### 2.3.2. Preparation of Refried Chicken Nuggets

The weighted chicken breast was cut into 1 cm^3^ pieces (the weighing was labeled M_0_) and combined with the composite coating powder. After a brief water soak (10 s) and 2 coatings (the weighing was labeled M_1_), the samples were fried at 170 °C for 4 min (the weighing was labeled M_2_), then cooled and freeze-dried at −40 °C for 48 h. The frozen pre-fried chicken pieces were refried at 180 °C for 1.5 min. M_0_, M_1_, and M_2_ were used to calculate the following coating pick-up and oil absorption ratio.

#### 2.3.3. Coating Pick-Up and Oil Absorption Rate

The pick-up of the chicken nuggets is defined as the ratio of the coating weight to the total chicken breast weight before coating:

Pick-up (%) = (M_1_ − M_0_)/M_0_×100%

where M_0_ is the chicken breast weight; M_1_ is the weight of the coated chicken breast.

The oil absorption rate is defined as the ratio of the oil absorption weight to the weight of the coated chicken breast.

Oil absorption ratio (%) = (M_1_ − M_2_)/M_1_×100%

where M_1_ is the weight of the coated chicken breast. M_2_ is the weight of the fried coated chicken breast.

#### 2.3.4. Texture Test

Texture properties were assessed using a CT3 Texture Analyzer (CT3 10K, Brookfield Instruments, Middlebury, USA). The specific parameters for the texture analysis were as follows: the TA11/1000 probe (cylindrical, 25.4 mm diameter and 35 mm long, and clear acrylic) was selected; the pretest, test, and return speeds for compression testing were all 1 mm/s; the load trigger force was 5 g; and the strain value was 50%. Each sample was tested five times. 

#### 2.3.5. Colors

The color of the samples was meticulously measured using a high-precision spectrophotometer (Chroma Meter CR-400, Koniva Minolta Sensinc, Tokyo, Japan) to obtain L*(Lightness), a*(red), b*(yellow), and △E values. Each group of samples was meticulously measured 6 times in parallel. L_0_*, a_0_*, and b_0_* referred to the color value of the whiteboard. △E was the total color difference.

#### 2.3.6. Sensory Evaluation

A sensory evaluation of the fried chicken nuggets was conducted using eight evaluators with a slight modification [6]. Before conducting the sensory evaluations, evaluators underwent standardized training to assess the sensory indicators. Evaluators were strictly prohibited from communicating with one another during the evaluation process. The color, crispness, surface structure, palatability (suitable greasiness), flavor, and comprehensive evaluation of the chicken nuggets were evaluated. Ratings varied from 0–10, with 0–4 being the worst, 5–7 moderate, and 8–10 best (Table 1). 

### 2.4. Data Analysis

Minitab software version 19 and Origin software version 8 software were used to analyze the experimental data and make charts, and each datum was repeated three times to obtain the average value.

## 3. Results and Discussions

### 3.1. Structure–Digestibility Analysis of Starch–Ethyl Maltol Samples

#### 3.1.1. Analysis of Complexation Ratio

**Figure 1:**
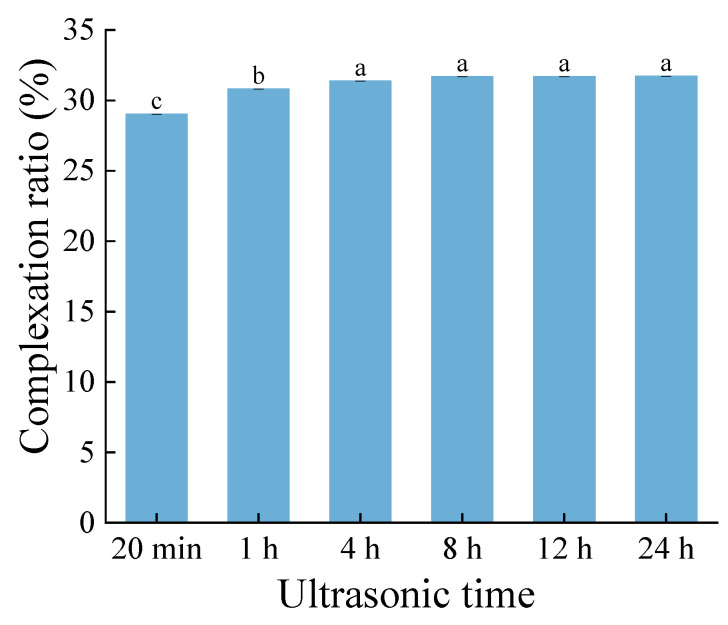
Complexation ratio at different ultrasonic times of the complex sample. Different letters indicate significantly different (*p* < 0.05).

The cavitation effect generated by ultrasonic waves can create a localized high-temperature and high-pressure environment in a very short time, disrupt the particle structure of starch [23], and consequently release the bound ethyl maltol. The ethyl maltol bound within the complex was released using ultrasonic treatment, and the complexation ratio of starch–ethyl maltol was assessed based on the amount of ethyl maltol released. After 20 min of ultrasonic treatment, the complexation ratio of ethyl maltol in the complex was approximately 29% (Figure 1). The release of ethyl maltol also exhibited a slight increase with prolonged ultrasonic exposure, reaching 30.8%. However, this release stabilized at 31.6% after 4 h of ultrasound treatment. Overall, the quantity of ethyl maltol released from the complex remained nearly constant with an extended ultrasonic treatment time. This also demonstrates the feasibility of preparing a starch–ethyl maltol complex using RCHMT, which enhances the flavor characteristics of powder-coated products.

#### 3.1.2. Analysis of Amylose Content 

As illustrated in Table 2, the corn starch has an amylose content of 31.77%. Upon the physical mixing of corn starch with ethyl maltol, a negligible reduction in amylose content is observed; however, this change is not statistically significant (*p* < 0.05). The amylose content in the corn starch–ethyl maltol complex exhibited a significant decrease (Table 2), which may be attributed to the formed hydrogen bonding between the amylose of corn starch and ethyl maltol, decreasing the available hydrogen bonding sites of starch. Consequently, during the determination of amylose content, this reduction in hydrogen bonding sites within the amylose complex diminishes the binding capacity of the iodine solution, ultimately resulting in a lower measured amylose content. 

#### 3.1.3. Morphology Observation

**Figure 2:**
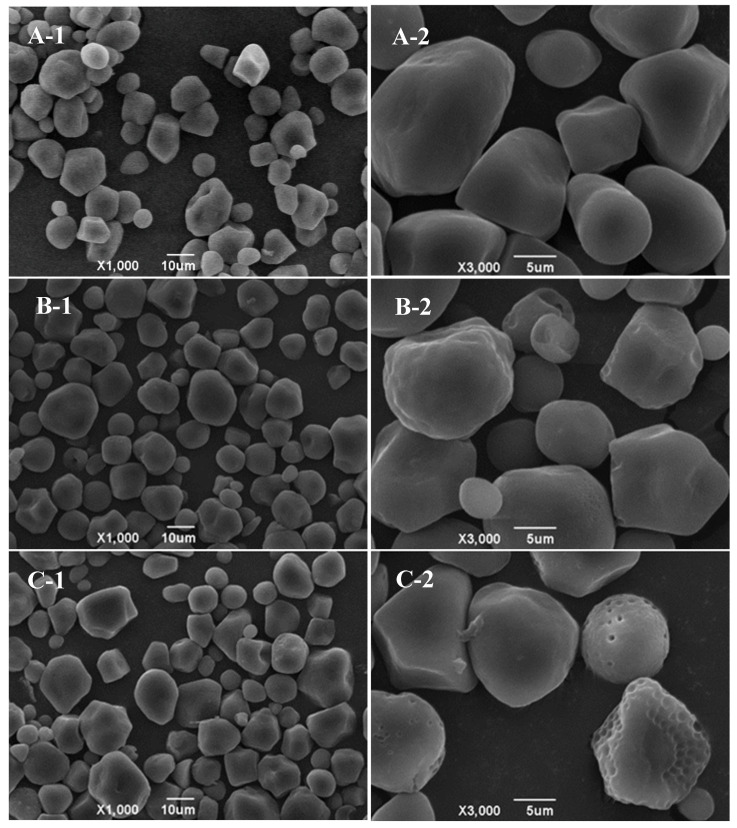
SEM pictures of native starch, mixture, and complex samples. (**A-1**,**A-2**) Native starch; (**B-1**,**B-2**) mixture sample; (**C-1**,**C-2**) complex sample; -1, 1000 magnification; -2, 3000 magnification.

The analysis in Figure 2 shows the diverse shapes of native corn starch, including balls, cylinders, ellipsoids, and irregular shapes characterized by a smooth granule surface (Figure 2(A-1,A-2)). When combined with ethyl maltol without RCHMT, the starch surface exhibits minimal indentation, preserving its overall structure (Figure 2(B-1,B-2)). When treated using RCHMT, the granule surface of the complex sample did not gelatinize and had dense pores, providing a pathway for ethyl maltol to enter the starch’s inner structure (Figure 2(C-1,C-2)). Red adzuki bean and rice starch present a coarse or indented granule surface and even present the aggregate phenomenon induced by heat moisture treatment [21,24]. However, the dense porous surface features of heat-moisture treatment have not been reported. This suggested that RCHMT may be a vital modification technique that makes it possible for ethyl maltol to enter the internal structure of starch. Generally, starch granules’ surface characteristics significantly impact the enzymatic desensitization and enzymatic hydrolysis mode of α-amylase, leading to substantial changes in starch digestibility [21].

#### 3.1.4. Long-Range Ordering Analysis

**Figure 3:**
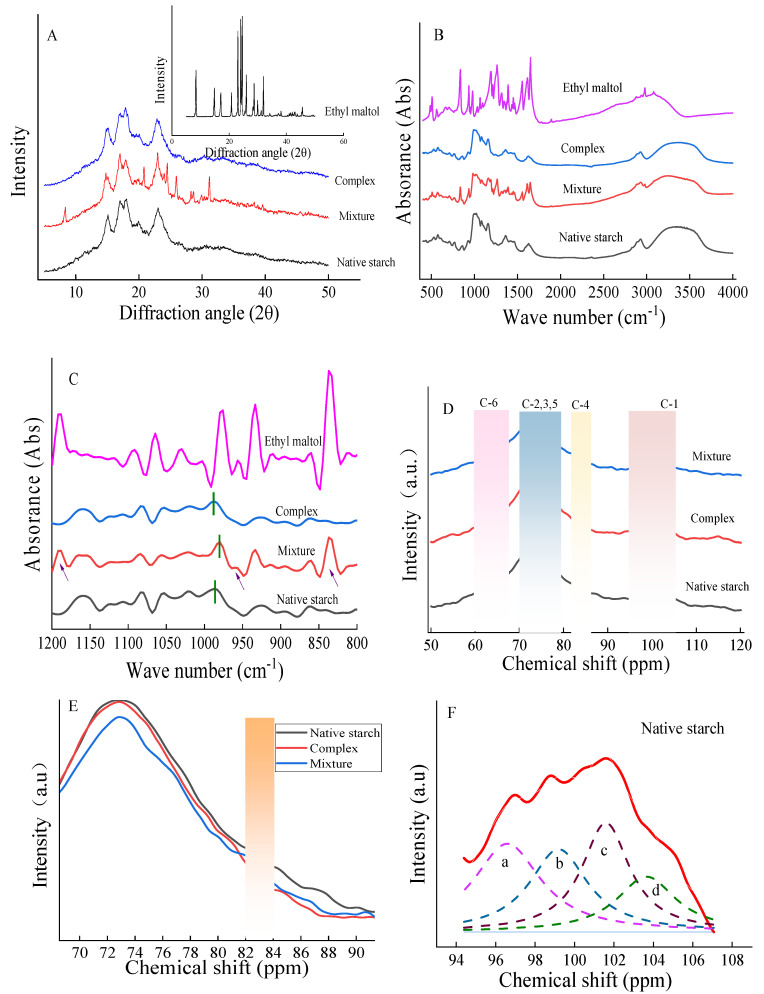

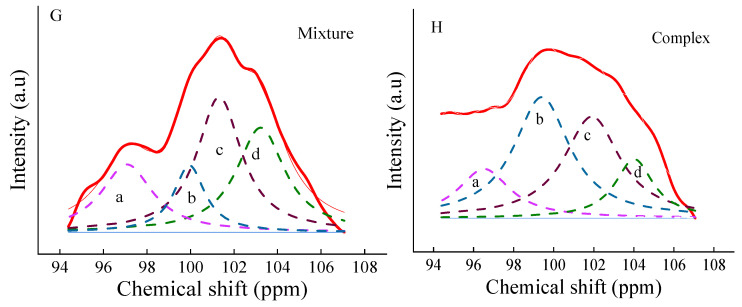
XRD patterns (**A**), infrared spectrum (**B**), infrared deconvolution (**C**), ^13^C-NMR spectra (**D**), locally enlarged image of ^13^C-NMR spectra (**E**), and fitting image in C1 regions of ^13^C-NMR spectra (**F**–**H**).

In Figure 3A, the distinctive diffraction pattern of ethyl maltol reveals narrow and prominent lines. Meanwhile, the diffraction pattern of native starch presents four sharp peaks at 2θ angles of 15°, 17°, 18°, and 23°, indicative of an A-type crystal structure [18].

Upon mixing starch with ethyl maltol without heat-moisture treatment, the observed crystal diffraction peak represents a superposition of both components (Figure 3A), likely due to the adsorption of ethyl maltol onto the surface of starch particles, allowing for the simultaneous detection of both substances.

The overall structure of the complex remained A-type (Figure 3A), with no peaks of ethyl maltol detected. There are two proposed mechanisms for the complexation of starch and ligands: one involves ligands entering the helical cavity structure of starch, resulting in the formation of an inclusion compound and a transition of the starch diffraction peak to a V-type crystal structure; the other describes an interaction between starch and ligands via non-covalent bonds, where the starch diffraction peak remains unchanged, leading to a non-inclusion compound [25]. In general, if ligands are not in the spiral cavity of starch, they form molecular aggregates with starch through hydrogen bonding, hydrophobic interaction, and electrostatic interaction [11]. Consequently, the complex’s original crystal structure (A-type) persisted even after the use of RCHMT, indicating that ethyl maltol combines with the external side of the starch straight chain through a non-covalent bond to form a non-inclusion complex. The RC, surface morphology of particles, and amylose content influence the gelatinization degree of starch, subsequently affecting the color, brittleness, and oil absorption characteristics of powder-coated products [7]. The higher RC of the complex than the mixture improves the quality of fired products (Table 2).

#### 3.1.5. Short-Range Ordering Analysis

Figure 3B and Table 2 show that the mixture increases the number of infrared absorption peaks compared to native starch. Fourier infrared deconvolution revealed new absorption peaks at wave numbers 1180, 958, and 895 cm^−1^ in the mixture. This suggests that the peaks of the mixture are the total superposition of starch and ethyl maltol. The new absorption peaks of ethyl maltol were absent in the complex. The characteristic peak of ethyl maltol was obscured in the infrared spectrum of the complex, which may be caused by amylose coating ethyl maltol through hydrogen bonding and other forces to form a starch–ethyl maltol complex. Other studies have also shown that the peaks of ligand molecules are masked after their inclusion in the host structure [26].

**Figure 4:**
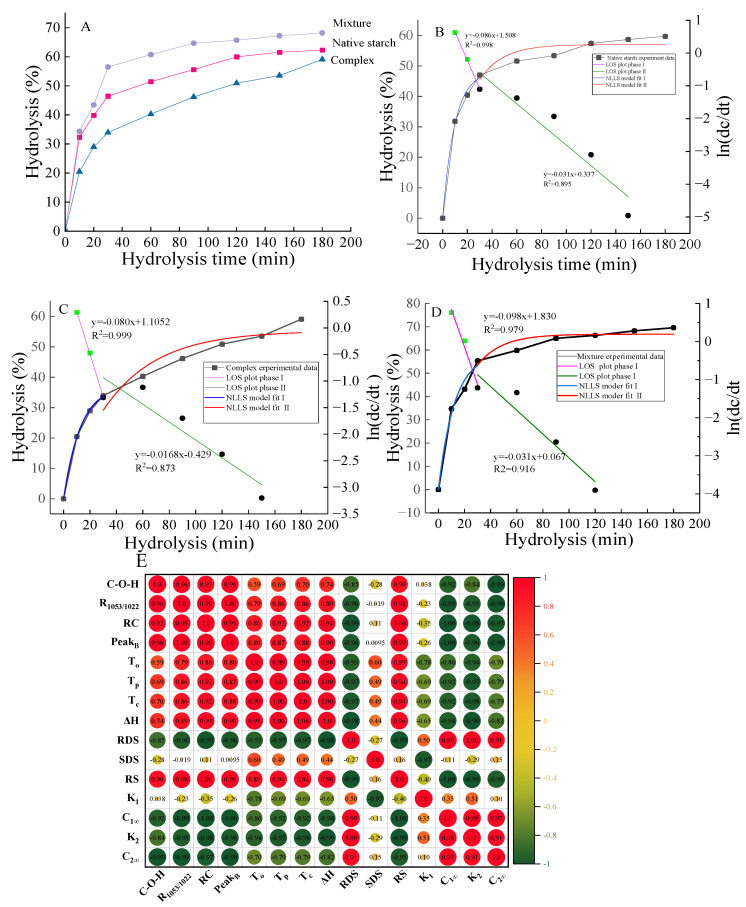
The hydrolysis curves (**A**), model fit curves, LOS plots (**B**–**D**), and correlation diagram (**E**) of native starch, mixture, and complex samples. E, Correlation analysis was performed using Pearson’s correlation analysis.

The second derivative was utilized to differentiate the infrared spectra of all samples, enabling the determination of the displacement of the C-O-H characteristic peak related to hydrogen bond formation at the C6 position of glucose units subsequent to starch dehydration. A blue shift in the C-O-H characteristic peak indicates an enhancement of hydrogen bonding within the system (Table 2), while a red shift signifies the weakening of hydrogen bonding [27]. The vibration absorption peak of C-O-H near 988 cm^−1^ is related to the hydrogen bond on C6 of the starch molecular chain and the dehydrated glucose unit [28]. The peak of the native starch was at 986 cm^−1^, and the peak of the mixture red-shifted to a lower wave number (980 cm^−1^) (Figure 3C), indicating an interference with the hydrogen bond structure of corn starch by ethyl maltol. Once the sample was treated using RCHMT, the -OH peak blue-shifted to a higher wave number (Table 2), showing that non-covalent bonds (hydrogen bonds) between starch and ethyl maltol were strengthened. Relative research confirms that the interaction between starch and phenolic acid results in a shift of the absorption peak [29]. R_1053/1020_ represents the short-range order of starch [17]. Following Fourier infrared deconvolution, shown in Table 2, the short-range order of the mixture decreased. At the same time, that of the complex increased, indicating that RCHMT helped to rearrange the crystal structure of the complex, enhancing the short-range order. The short-range order also significantly correlated with starch resistance to digestion (Figure 4E).

#### 3.1.6. ^13^C Solid-State NMR Analysis

^13^C solid-state NMR analysis can provide valuable information about the molecular structure of a sample by revealing changes in the chemical shifts. Nuclear magnetic resonance spectroscopy categorizes chemical shifts into four regions (C_1_, C_4_, C_2, 3, 5_, and C_6_) based on the properties of the carbons (Figure 3D). Peak resonances at 60–67, 70–79, 82–84, and 94–105 ppm are associated with C_6_, C_2,3,5_, C_4_, and C_1_ carbons, respectively [30]. Specifically, peaks around 103 ppm correspond to the amorphous region of starch, peaks near 100 ppm are linked to a double-helix or quasicrystal structure, and peaks at 96.7 ppm and 94.5 ppm are connected to less-favorable configurations of starch [31]. In the C_4_ region, the peak intensities can serve as indicators of changes in the amorphous composition of starch, and a stronger signal in the C_4_ region suggests a higher content of the amorphous region [32]. In Figure 3E, the peak intensity of the C_4_ region of native starch is the greatest, indicating a larger proportion of amorphous regions. Moreover, the peak intensity in the 82–84 ppm range was weaker than that of the native starch and the mixture (Figure 3E), indicating that the proportion of the amorphous zone decreased due to RCHMT.

To understand the interaction between ethyl maltol and starch after RCHMT, the focus was on the peak treatment of the C_1_ region (Figure 3F–H). Analysis revealed three distinct peaks between 100 and 105 ppm in the C1 region of both the native starch and the complex, indicating that the sample formed an A-type double-helix structure after three refolds, which aligns with the crystal structure findings from XRD (Figure 3A). Although the mixture also exhibits three peaks, in conjunction with the XRD results, it can be concluded that the mixture consists of superimposed peaks from starch and ethyl maltol. Additionally, the results in Table 2 demonstrate that the order of peak area near 100 ppm (Peak_b_) is complex > native starch > mixture, showing that the crystallinity of the complex improves after RCHMT, in line with the RC results (Table 2). The Peak_b_ area has a highly positive correlation with RC in Figure 4E.

#### 3.1.7. Thermal Properties Analysis

The phase transition starting temperature is denoted as T_o_, the peak temperature is T_p_, and the final value temperature is T_c_. The peak temperatures of corn starch and ethyl maltol are 74.5 °C and 93.1 °C, respectively, which are lower than that of the starch–ethyl maltol complex. This indicates the presence of non-covalent bonds between starch and ethyl maltol, which enhance the crystal structure of starch and necessitate a higher temperature for gelatinization (Table 3). A positive relationship between hydrogen bonds and thermal parameters is presented in Figure 4E.

Gelatinization enthalpy (ΔH) is associated with the double-helix structure of starch and its crystal state [20]. The complex exhibited larger gelatinization enthalpy compared to the mixture due to the presence of intermolecular interactions. This implies that more energy is required to disrupt its spiral structure [17], indicating a relatively strong compound effect. In general, the thermal stability of the complex was greatly improved, that is, the ethyl maltol required higher energy to be released. ΔH had a positive correlation with the RC, Peak_b_ area, and RS content, and a negative correlation with the hydrolysis parameter (Figure 4E). Starch exhibiting enhanced thermal stability is less susceptible to gelatinization, thereby contributing to the preservation of the crispness of the fried food’s coating [6], which also provides ideas for the development of products with high crispness.

#### 3.1.8. Digestive Characteristics Analysis

Table 3 shows the results of RDS, SDS, and RS. The RS content in the mixture decreased compared to native starch. This could be due to ethyl maltol interfering with the short-range order of the starch, making it easier to break down by the enzyme amylase. Figure 4E also proves that RS has a positive correlation with short-range order and a negative correlation with the hydrolysis parameters. The RS content of the complex significantly increased after RCHMT. Generally, the microporous structure or degradation of starch granules helps increase the sensitivity of amylase and improve starch digestibility [33,34]. In theory, the porous structure of the surface of the complex sample helped to improve the digestibility, but the results contradict this, further confirming that the hydrogen bond formed between corn starch and ethyl maltol strengthens the starch’s structure and reduces the enzyme sensitivity. In the combination of starch–ferulic acid, the strengthened hydrogen bond between the two compounds was also confirmed to reduce the digestibility of amylase [35]. Therefore, regulating moisture and temperature in food processing to combine food raw materials with ligands (fat, flavor substances, polyphenols, etc.) is an important research direction for reducing the digestibility of starch and regulating blood sugar.

#### 3.1.9. First-Order Kinetic Analysis

The LOS model is used to identify multiple linear phases in the digestion process and determine the phase change time. The NLLS model is then fitted to obtain the actual values of K, and the C_∞_ LOS fitting results indicate that the rapid digestion stage of starch hydrolysis occurs from 0 to 30 min (Stage I), followed by a slower hydrolysis rate in Stage II. C_1∞_ and C_2∞_ represent the hydrolysis rates at the endpoints of Stage I and Stage II, respectively. K_1_ and K_2_ correspond to the enzymatic hydrolysis rates for Stage I and Stage II, respectively. A higher K value signifies a faster digestion rate. Stage I is hydrolyzed faster, and this part of starch can rapidly increase the blood sugar content in the human body. Due to its stress on the blood sugar regulation system, it can cause many diseases, while Stage II is milder and conducive to disease prevention. Two sets of first-order kinetic constants (K_1_, K_2_) and percentage hydrolysis (C_1∞_, C_2∞_) are shown in Table 3. Consistently, the K value of the first phase surpassed that of the second phase, attributable to the removal of quickly digestible starch during rapid hydrolysis, leaving only starch less sensitive to the enzyme [36]. Additionally, the K value and C_∞_ of the complex sample were notably smaller than those of the native starch and mixture (Table 3). The presence of ethyl maltol tightened the internal structure through hydrogen bonding, reducing the enzyme sensitivity of the complex starch, similar to the effects observed in the complex of starch and polyphenols [37].

Furthermore, studies have shown that porous starch granules enhance overall digestibility by allowing enzymes to interact with the surface structure [33]. Despite the porous structure of the complex, this did not lead to improved enzyme digestibility. The reduced starch digestion in the complex may be attributed to the non-covalent interaction of the starch molecular structure in the presence of ethyl maltol, altering the hydrogen bonds of the starch chains and ultimately stabilizing and reducing glucose release. Therefore, the advantages of the lower hydrolysis rate and enzymatic hydrolysis rate of the complex also provide an important research direction for the development of functional fried products to reduce blood sugar.

### 3.2. Application of Complex to Pre-Fried Chicken Pieces

#### 3.2.1. Coating Pick-Up and Oil Absorption Rate of Chicken Nuggets Prepared with the Complex with Different Addition Ratios

**Figure 5:**
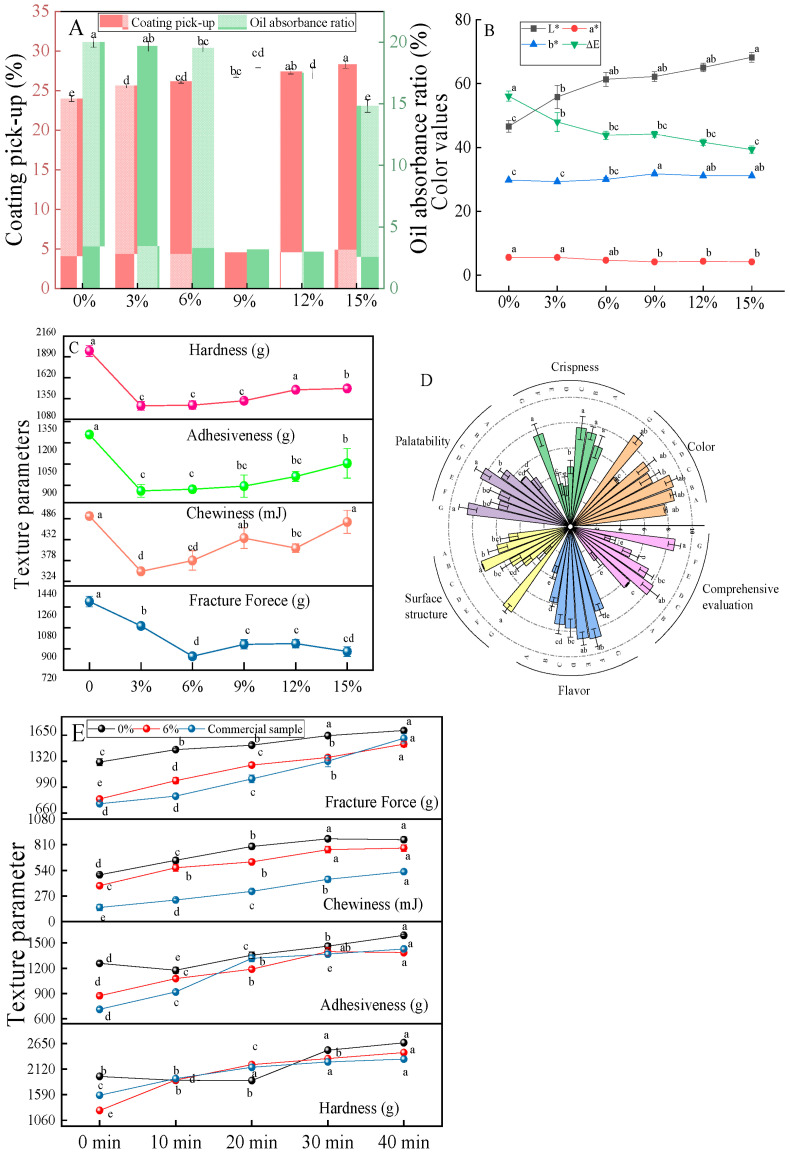
Coating pick-up (**A**), oil absorption ratio (**A**), color values (**B**), texture (**C**), rose diagram of artificial sensory evaluation (**D**) of the chicken nuggets prepared using the complex with different addition ratios, and the texture (**E**) of chicken nuggets fried at different times. In the rose diagram of the artificial sensory evaluation (**D**), the letters A–F are the samples prepared by the complex with different addition ratios (0%, 3%, 6%, 9%, 12%, and 15%), and the letter G is the commercial sample. The lowercase letters are significantly different (*p* < 0.05).

Coating pick-up is an important indicator for fried food, affecting its quality and production rate. The main components of traditional coating powder are wheat flour and starch [1]. The impact of this complex on the coating pick-up was evaluated. Without the addition of the complex, the coating pick-up of the sample was 23.8% (Figure 5A). As the complex was added, the thickness slightly increased, with no significant difference when the complex addition ratio interval was 3%, but a significant difference when the ratio interval was 6% (Figure 5A). Generally, amylopectin is prone to water absorption and swelling [38]. The addition of the complex increased the coating pick-up, indicating that the complex powder contained a higher amylopectin content. This was because amylose was combined with ethyl maltol during the RCHMT process, resulting in a decrease in amylose content and an increase in amylopectin content. The lower amylose content also confirmed the results in Table 2.

The oil absorbance ratio is an important indicator for evaluating the quality of fried products. The structure and properties of starch are key factors determining fried food’s oil absorption and texture. The variation in starch oil absorption is related to the granule morphology, crystalline properties, double-helix structure, and molecular properties of starch [39]. When no complex was added, the oil uptake was high, but once the complex was introduced, the oil uptake was significantly reduced (Figure 5A). Theoretically, the surface pore structure could increase oil absorption [40], but the oil uptake decreased in reality. Therefore, the introduction of ethyl maltol affects the hydrophobic structure in starch, preventing oil absorption. Meanwhile, corn starch and ethyl maltol interact through hydrogen bonding and other forces to enhance the crystal structure of starch. Additionally, moisture and temperature during heat-moisture treatment increase the rearrangement of starch molecular chains, further strengthening its structure and forming a dense, orderly crystal that hinders oil entry. The addition of resistant starch can also reduce oil uptake [41]. Therefore, the addition of starch with a high RS content in industrial product processing is helpful to reduce the final oil absorption rate of the product.

#### 3.2.2. Colors of Chicken Nuggets Prepared Using the Complex with Different Addition Ratios

Color is a crucial element for determining the perceived quality of food. When subjected to high-temperature frying, starch undergoes various physical and chemical changes, such as dehydration, starch gelatinization, protein denaturation, and the Maillard reaction [42]. These changes significantly impact the oil absorption ratio and color transformation. With an increase in the proportion of the complex sample, the chicken nuggets showed a decrease in the a* value and an increase in the L* and b* values (Figure 5B), resulting in a yellowish-brown appearance due to heightened color absorption from pigments generated by the Maillard and caramelization reactions [6]. The overall color difference may stem from temperature and moisture content changes, triggering non-enzymatic browning, like the Maillard reaction and caramelization [43]. Compared with the native starch, the total color value exhibited a significant change post-compound addition (Figure 5B). However, the total color difference did not change with the addition of the complex. This emphasized the complex’s capacity to reliably transform the product’s color. It was observed that heat-moisture treatment facilitated the thermal degradation of starch, leading to the cleavage of both α,1-4 and α,1-6 glucosidic bonds [44]. It was speculated that the starch molecular degradation during complex preparation likely resulted in the increased leakage of glycosidic bonds, which then reacted with the wheat flour protein in the coating through the Maillard reaction, causing the color of the chicken nuggets to intensify with the addition of the complex.

#### 3.2.3. Texture of the Chicken Nuggets Prepared Using the Complex with Different Addition Ratios

Texture is a vital aspect of quality measurement, and crispness is a highly desirable trait. Different proportions of complexes were added to the chicken nuggets to detect the effect on the product’s texture. The hardness, adhesiveness, and chewiness increased as the complex proportion increased, while the fracture force decreased overall, especially at 6% (Figure 5C). Some researchers have positively linked hardness with crispness [2], while others have discovered an inverse relationship between hardness and crispness [45]. From a sensory perspective, hardness and crispness are distinct sensory properties. A sample with greater hardness is more challenging to break, while a sample with greater crispness is more likely to break. Fracture force is commonly used to indicate brittleness, representing the peak of the TPA force with the first breaking point on the distance curve. A lower fracture force indicates a crisper specimen [6]. The fracture force initially decreased and then increased with an increase in the complex proportion. Hence, 6% is the crucial number for achieving the perfect crispy chicken nuggets. Starch-ethyl maltol has high thermodynamic stability and is not prone to rapid gelatinization during frying, thus forming a crispy coating [6].

#### 3.2.4. Sensory Evaluation of Chicken Nuggets Prepared Using the Complex with Different Addition Ratios

As the complex proportion increased from 0% to 12%, no significant visual differences were observed; however, an unpleasant dark color appeared at a 15% ratio (Figure 5D). The greater the proportion of complex added, the more difficult it is for the crispness to be accepted by consumers. However, as the proportion of the complex was increased, the meat flavor showed an increasing trend, mainly due to the higher proportion of complex containing more ethyl maltol (Figure 5D). At the same time, the comprehensive sensory scores of the commercial products had no statistical difference compared to those of the chicken nuggets with a 6% complex addition (Figure 5D). The best palatability was achieved at a compound powder ratio of 9%. Higher amounts of added powder lead to higher oil absorption and poorer palatability, while lower amounts reduce oil absorption, leading to a lack of smell saturation created by the oil. Overall, the quality of commercially available chicken nuggets is comparable to that of the complex products.

#### 3.2.5. Comparison of the Texture of Chicken Nuggets Fried for Different Times

A common issue in the market is that chicken nugget products quickly lose their crispiness after frying, diminishing the sensory experience for consumers and limiting the growth of deep-fried products. A study was conducted to analyze the texture changes in chicken nuggets with an optimal complex addition ratio compared with the control sample and commercial products. The results reveal that the complex sample can effectively regulate the crispiness of the chicken nuggets for an extended period (Figure 5E), matching the quality of commercially available products. Enhanced hydrogen bonding between starch and ethyl maltol likely contributes to a long-lasting crispy texture. This unique combination makes the starch resistant to enzymatic breakdown and boosts the resistant starch content. Resistant starch has better expansibility and solubility, making the batter puffier and crisper after frying [41]. This breakthrough enables us to offer products that maintain their crispness over time, ultimately enhancing the consumer’s experience and driving the rapid development of deep-fried coated products.

## 4. Conclusions

The study aimed to prepare a corn starch-ethyl maltol complex using RCHMT, with native starch and a corn starch-ethyl maltol mixture as controls. The results show that the complexation ratio of ethyl maltol reaches about 31.6%. Additionally, a microporous structure was formed on the complex’s surface. Analyses of the crystal structure and peak fitting in the C_1_ region revealed that the complex had an A-type crystal structure, with the amorphous region of the complex being the smallest. FT-IR results show that hydrogen bond positions in the complex are blue-shifted, but the mixture shows a red shift. The thermal parameters of and resistant starch in the complex increased, and the hydrolysis was divided into two stages by LOS fitting. In conclusion, a non-inclusion complex was formed by starch and ethyl maltol after using RCHMT, mainly through the hydrogen bond structure formed by amylose and ethyl maltol. Subsequently, different proportions of the complex were added to chicken nuggets, and a 6% addition resulted in the highest crispness, best palatability, and a better sensory score. In comparison with commercial chicken nuggets, the overall texture was found to be comparable. Therefore, RCHMT can provide a new method for synthesizing a stable corn starch flavor complex. The results of this study lay the foundation for analyzing the interaction mechanism between corn starch and flavor substances, and also provide ideas and references for the development of powder-coated products.

## Figures and Tables

**Table 1:** Sensory evaluation indicators of chicken nuggets with different proportions of the complex.

Scores	Color	Crispness	Surface Structure	Palatability	Flavor	Comprehensive Evaluation
8–10	Golden yellow, uniform color	Crisp, moderately hard, no sticking	Fish scale, moderate thickness of coating powder	Appropriate oil content	Full meat aroma, no pungent taste	Favorite
5–7	Light yellow	Medium crispy, harder or softer	Poor fish scale, high or low thickness of coating powder	Medium suitable greasiness	Faint meat aroma	General
0–4	Dark color, uneven color	Too hard or too soft	Too thick or too thin coating powder	Excessive greasiness	Meatless flavor	Unacceptable

**Table 2:** Amylose content; the wave band of C-O-H def., CH2; ratio value of 1053/1020; relative crystallinity and ^13^C-NMR parameters of native starch, mixture, and complex samples *.

Samples	Amylose Content	C-O-H def., CH2	R_1053/1020_	RC	Peaks	Chemical Shift (ppm)	Area Ratio (%)
Native starch		986	1.96 ± 0.04 ^b^	20.06 ± 0.01 ^a^	Peak_a_	96.5	27.44
	31.77 ± 0.21 ^a^				Peak_b_	99.1	26.10
					Peak_c_	101.5	29.39
					Peak_d_	103.6	17.06
Mixture		980	1.29 ± 0.03 ^c^	18.45 ± 0.01 ^c^	Peak_a_	97.7	19.56
					Peak_b_	99.9	13.32
	31.47 ± 0.07 ^a^				Peak_c_	101.3	36.98
					Peak_d_	103	30.12
Complex		988	2.60 ± 0.02 ^a^	22.50 ± 0.01 ^b^	Peak_a_	96.5	13.54
					Peak_b_	99.4	39.58
	26.29 ± 0.08 ^b^				Peak_c_	101.9	33.38
					Peak_d_	104.0	13.33

* Data in a column with different letters are significantly different (*p* < 0.05).

**Table 3:** Thermal parameter, starch components, and hydrolysis parameter of native starch, mixture, and complex samples *.

	Parameters	Native Starch	Mixture	Complex	Ethyl Maltol
Thermal parameters	T_o_ (°C)	65.05 ± 1.77 ^c^	66.35 ± 0.07 ^c^	74.30 ± 0.42 ^b^	84.86 ± 0.25 ^a^
	T_p_ (°C)	74.55 ± 0.50 ^c^	74.55 ± 0.35 ^c^	108.32 ± 0.12 ^a^	93.10 ± 0.42 ^b^
	T_c_ (°C)	77.40 ± 0.71 ^c^	76.98 ± 0.30 ^c^	115.69 ± 0.20 ^a^	108.16 ± 0.25 ^b^
	ΔH/(J/g)	9.28 ± 0.07 ^c^	8.97 ± 0.09 ^c^	13.31 ± 0.16 ^a^	11.61 ± 0.10 ^b^
Starch components	RDS	39.83 ± 1.15 ^b^	43.42 ± 0.56 ^a^	28.97 ± 1.32 ^c^	
	SDS	21.67 ± 1.40 ^a^	23.14 ± 0.53 ^a^	23.13 ± 0.33 ^a^	
	RS	38.5 ± 0.31 ^b^	33.44 ± 0.84 ^c^	47.9 ± 1.11 ^a^	
Hydrolysis parameters	K_1_	0.090 ± 0.001 a	0.081 ± 0.006 ^a^	0.078 ± 0.006 ^a^	
	C_1∞_	50.08 ± 1.110 ^b^	58.67 ± 3.071 ^a^	37.33 ± 1.590 ^c^	
	K_2_	0.037 ± 0.006 ^a^	0.041 ± 0.010 ^a^	0.024 ± 0.001 ^a^	
	C_2∞_	60.05 ± 1.680 ^b^	67.88 ± 0.094 ^a^	55.32 ± 0.153 ^c^	

* Data in a line with different letters are significantly different (*p* < 0.05).

## Data Availability

The original contributions presented in this study are included in the article/Supplementary Materials. Further inquiries can be directed to the corresponding author.

## Supplementary Materials

The following supporting information can be downloaded at: https://www.mdpi.com/article/10.3390/foods13223629/s1, Figure S1: (A–E) The influence of single factor condition on complexation ratio of repeated-continuous heat-moisture treatment; Table S1: Response surface analysis scheme and experimental results. Figure S2: (A–C) 3D diagram of the effect of interaction between three factors on recombination rate; Table S2: Regression model analysis of variance.
