# Supplementary material for: Interactions Between Corn Starch and Ethyl Maltol Under Heat-Moisture Treatment and Its Application in Fried Chicken Nuggets
Source: Foods. 2024 Nov 14;13(22):3629. doi: 10.3390/foods13223629 (PMC11593958; doi:10.3390/foods13223629)
Supplement: Supplementary file 1 [file foods-13-03629-s001.zip › foods-3268579-supplementary.pdf]

foods

Article

# Interactions Between Corn Starch and Ethyl Maltol Under Heat-Moisture Treatment and Its Application in Fried Chicken Nuggets

**Meijuan Xu [1,2], Tianwen Liu [3], Xueqin Gao [1], Yuran Shi [1], Xiaodong Zhao [1] and Jian Zou [1,*]**

1 College of Food and Biological Engineering, Henan University of Animal Husbandry and Economy, Zhengzhou 450046, China; xumeijuan113@163.com (M.X.); kfgxq03@163.com (X.G.); shiyuran0907@163.com (Y.S.); 15093306674@163.com (X.Z.)
2 College of Food Science and Technology, Huazhong Agricultural University, Wuhan 430070, China
3 School of Life and Health Sciences, Hubei University of Technology, Wuhan 430068, China; ltw15515757402@163.com
* Correspondence: 81450@hnuahe.edu.cn

S1. Analysis of single-factor and response surface analysis

The impact of water content, ethyl maltol content, treatment temperature, treatment time, and treatment cycles on the recombination rate was investigated. The results show that water content, treatment temperature, and treatment time significantly affect the complexation ratio (Figure S1).

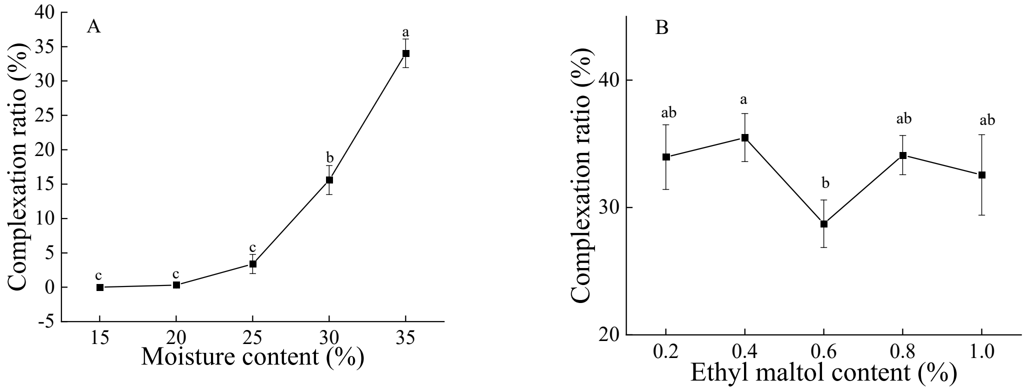

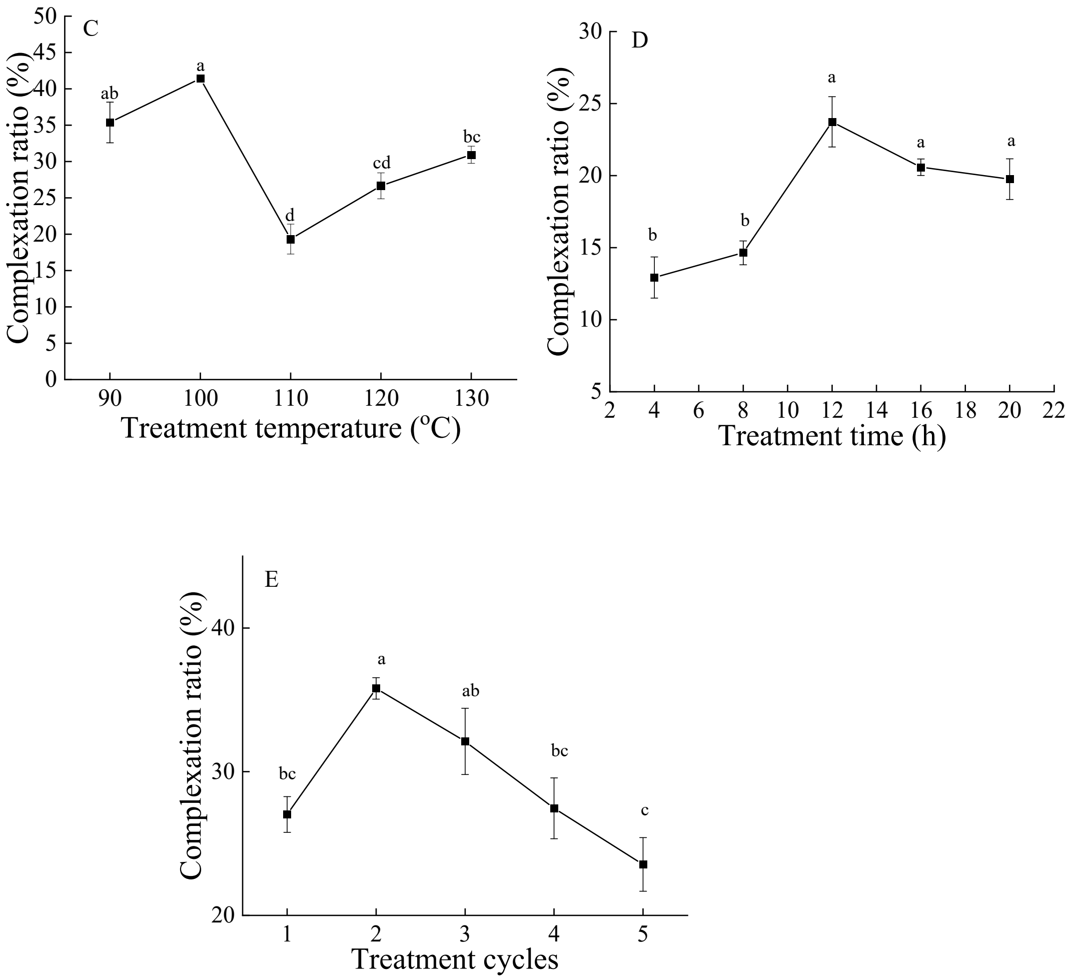


**Figure S1.** (**A–E**) The influence of single factor condition on complexation ratio of repeated-continuous heat-moisture treatment. Different letters are significantly different ($p < 0.05$).

Based on the single-factor test, the three single factors (treatment temperature, water content, treatment time) that have the greatest influence on the complexing ratio of corn starch complex were selected to investigate the influence of interaction on the recombination rate. The experimental design scheme and results are shown in Table S1. The regression curve of corn starch was: Y2=31.88-2.55A+8.41B+1.49C-0.9650AB+0.17AC+0.02BC-8.68A2-9.15B2-6.51C2. The results of the variance analysis of the regression model are shown in Table S2. It can be seen that the response surface model of the corn starch complex is significant ($p<0.01$). The missing item was not significant ($p=0.9639$, not significant at the 0.05 level), indicating a good fit for the equation. The coefficient of determination $R^2=0.9753$ shows a strong correlation between actual and predicted values.

**Table S1.** Response surface analysis scheme and experimental results [a]

| Number | Factors | | | Complexing ratio/% |
|---|---|---|---|---|
| | A | B | C | |
| 1 | 100 | 30 | 12 | 34.4 |
| 2 | 100 | 30 | 12 | 27.15 |
| 3 | 90 | 35 | 12 | 25.38 |
| 4 | 110 | 25 | 12 | 4.66 |
| 5 | 100 | 30 | 12 | 34.73 |
| 6 | 100 | 30 | 12 | 32.15 |
| 7 | 100 | 25 | 8 | 6.46 |
| 8 | 90 | 30 | 16 | 21.29 |
| 9 | 110 | 30 | 16 | 15.52 |
| 10 | 100 | 35 | 8 | 23.43 |
| 11 | 90 | 30 | 8 | 18.22 |
| 12 | 100 | 35 | 16 | 26.03 |
| 13 | 110 | 30 | 8 | 11.77 |
| 14 | 90 | 25 | 12 | 6.84 |
| 15 | 100 | 25 | 16 | 8.98 |
| 16 | 100 | 30 | 12 | 30.99 |
| 17 | 110 | 35 | 12 | 19.34 |

[a] A, B and C indicate the treatment temperature ($^oC$), water content (%) and treatment time (h).

Therefore, the model can reflect the relationship between each factor and the response value and predict the best process conditions. According to the P value, the influence of moisture B on the recombination rate was extremely significant ($p<0.01$), the temperature of humid heat treatment was significant ($p<0.05$), and the rest were not significant. According to the F value, the order of influence of the three factors on the recombination rate is B>A>C, that is, water content > treatment temperature > treatment time.

**Table S2.** Regression model analysis of variance [a,b]

| Sour | Sum of squares | df | Mean square | F-value | P-value | Significant |
|---|---|---|---|---|---|---|
| Model | 1583.95 | 9 | 175.99 | 30.68 | < 0.0001 | ** |
| A | 52.22 | 1 | 52.22 | 9.10 | 0.0195 | * |
| B | 565.15 | 1 | 565.15 | 98.53 | < 0.0001 | ** |
| C | 17.82 | 1 | 17.82 | 3.11 | 0.1213 | |
| AB | 3.72 | 1 | 3.72 | 0.6494 | 0.4468 | |
| AC | 0.1156 | 1 | 0.1156 | 0.0202 | 0.8911 | |
| BC | 0.0016 | 1 | 0.0016 | 0.0003 | 0.9871 | |
| $A^2$ | 317.01 | 1 | 317.01 | 55.27 | 0.0001 | ** |
| $B^2$ | 352.67 | 1 | 352.67 | 61.48 | 0.0001 | ** |
| $C^2$ | 178.28 | 1 | 178.28 | 31.08 | 0.0008 | ** |
| Residual | 40.15 | 7 | 5.74 | | | |
| Lack of fit | 2.44 | 3 | 0.8138 | 0.0863 | 0.9639 | |
| Pure Error | 37.71 | 4 | 9.43 | | | |
| Cor Total | 1624.10 | 16 | | | | |
| $R^2$ | 0.9753 | | | | | |
| $R^2$ Adj | 0.9435 | | | | | |

[a] A, B and C indicate the treatment temperature (°C)、water content (%) and treatment time (h).

[b] * indicates a significant difference ($p$<0.05); ** indicates a very significant difference ($p$<0.01).

The study evaluated the optimal recombination rate process under the combined influence of heat-moisture treatment temperature, water content, and continuous heat-moisture treatment time (Figure S2). The predicted recombination rate was 31.07% under heat-moisture treatment conditions with a temperature of 94.15°C, moisture at 34.40%, and continuous heat treatment for 11.7 hours. When the heat treatment temperature was 94°C, water content was 34.4%, and continuous treatment time was 11.7 hours, the measured recombination rate was 29.01%. The predicted values are close to the measured ones, indicating that the model can be used to optimize the process of corn starch-ethyl maltol complex.

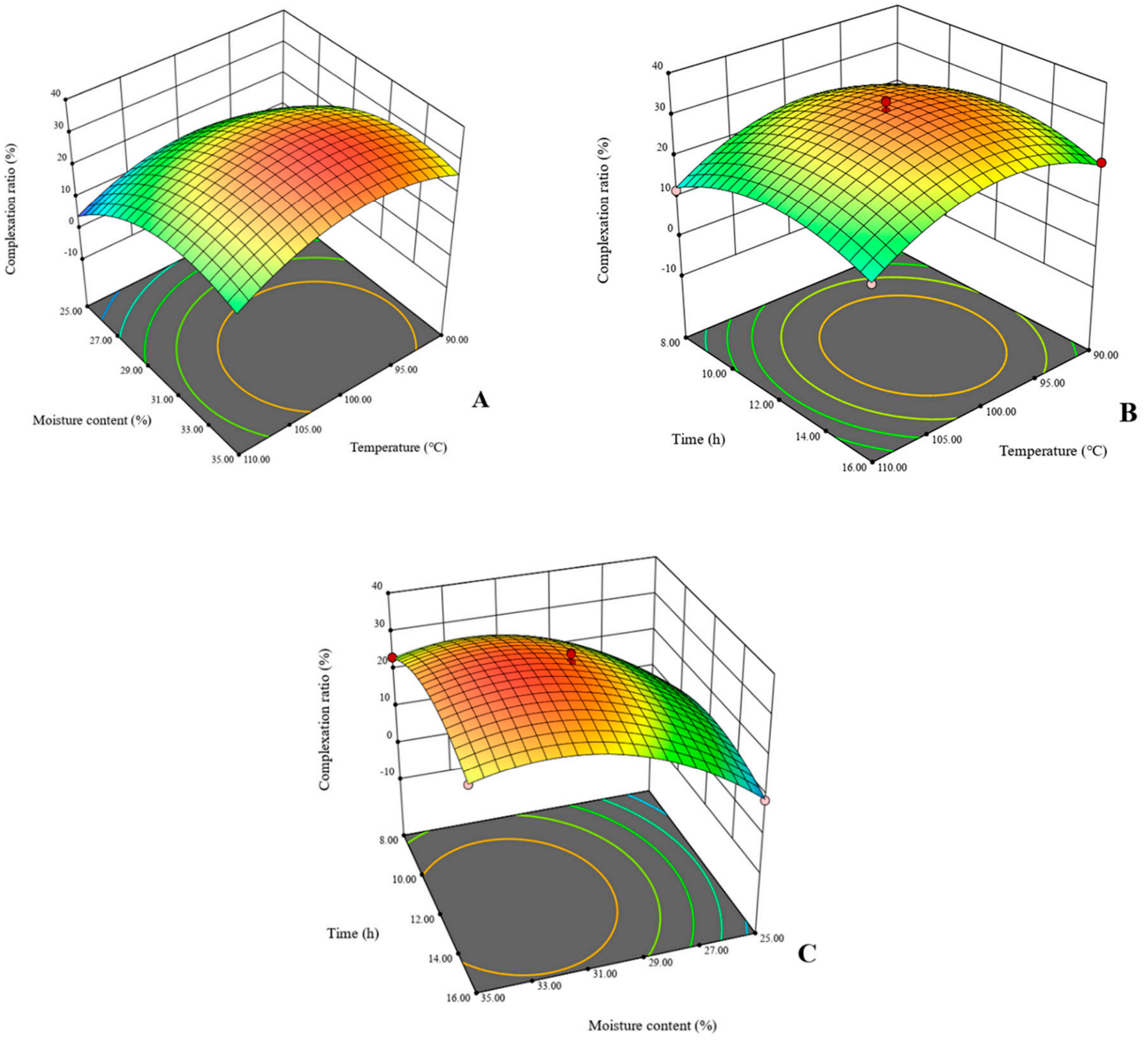


**Figure S2.** (**A–C**) 3D diagram of the effect of interaction between three factors on recombination rate
